# Inflammatory biomarkers prior to antiretroviral therapy as prognostic markers of 12-month mortality in South Africa and Uganda

**DOI:** 10.1097/QAD.0000000000002305

**Published:** 2019-07-02

**Authors:** Mark J. Siedner, Mwebesa Bosco Bwana, Stephen Asiimwe, Nicholas Musinguzi, Jose Castillo-Mancilla, Gideon Amanyire, Russell P. Tracy, David R. Bangsberg, Catherine Orrell, Jessica E. Haberer

**Affiliations:** a Harvard Medical School; b Massachusetts General Hospital, Boston, Massachusetts, USA; c Mbarara University of Science and Technology, Mbarara, Uganda; d Africa Health Research Institute, Kwa-Zulu Natal, South Africa; e University of Colorado, Denver, Colorado; f University of Vermont, Burlington, Vermont; g Oregon Health Sciences University-Portland State University School of Public Health, Portland, Oregon, USA; h University of Cape Town, Cape Town, South Africa.

**Keywords:** antiretroviral therapy, HIV, inflammation, mortality, South Africa, Uganda ;

## Abstract

The aim of this study was to determine the utility of biomarkers of immune activation, systemic inflammation and coagulopathy prior to antiretroviral therapy to predict mortality during the first year of antiretroviral therapy (ART) in sub-Saharan Africa.

**Design:**

A prospective, observational cohort.

**Methods:**

We measured soluble CD14, interleukin-6 and D-dimer in nonpregnant individuals initiating ART in South Africa and Uganda in the Measuring Early Treatment Adherence (META) Study. We used survival analysis methods to estimate their association with 12-month mortality, and fit receiver operator curves (ROC) to assess the prognostic value of each biomarker.

**Results:**

Six-hundred and sixty individuals were enrolled and had pretreatment biomarkers measured. Approximately 60% were women, with a median CD4^+^ cell count of 187 cells/μl [interquartile range (IQR) 111–425] and approximately half were enrolled each from South Africa and Uganda. We observed 34 deaths for a crude mortality of 5.3 deaths/100 person-years (py) (95% confidence interval 3.8–7.4), which ranged from 0/100 py to 13.7/100 py in the lowest and highest tertile of pretreatment sCD14, respectively. In Cox models, all three biomarkers were strongly predictive of the hazard of death (adjusted hazard ratio 3–6, all *P* < 0.01). In multivariable models including biomarkers, both pretreatment CD4^+^ cell count and pretreatment viral load became borderline or nonsignificantly associated with mortality. The c-statistic for area under ROC was higher for all three biomarkers than for CD4^+^ cell count (*P* < 0.01).

**Conclusion:**

Biomarkers of immune activation, systemic inflammation and coagulopathy prior to ART initiation are strongly predictive of early death on treatment after adjustment for CD4^+^ cell count. Such biomarkers might serve as important prognostic indicators for patient triage in this population.

## Abstract 2

Supplemental Digital Content is available in the text

## Introduction

Presentation to care with advanced HIV persists in sub-Saharan Africa (SSA) [1–3], despite efforts to start antiretroviral therapy (ART) regardless of disease stage [4]. Indeed, although AIDS mortality is declining in the region, approximately 25% of people continue to present with a CD4^+^ cell count less than 200 cells/μl, and mortality rates remain between 5 and 10% early during treatment [5,6].

In the face of persistently high mortality, and in the context of expanding ART programmes with limited resources, many treatment programmes are adopting differentiated models of care [7–9]. These programmes prioritize human and financial resources for patients at highest risk of mortality and loss from care, while simultaneously permitting stable patients to have less frequent interface with the health system. To be successful, this strategy requires development of simple and scalable tools to differentiate between these patient populations.

Biomarkers of immune activation, systemic inflammation and coagulopathy have been previously associated with future risk of opportunistic infections, noncommunicable disease and all-cause mortality [10–14]. We sought to assess the utility of potentially scalable blood tests of systemic inflammation, immune activation and coagulopathy for prediction of mortality in a prospectively observed longitudinal cohort of individuals in routine programmatic care in South Africa and Uganda in the current era of ART.

## Materials and methods

### Study setting and participants

Study participants were enrolled in the Monitoring Early Treatment Adherence Study during March 2015–September 2016 (META, NCT02419066). The study had a primary goal to determine whether disease stage [early (CD4^+^ cell count >350 cells/μl) versus late (CD4^+^ cell count <200 cells/μl)] or pregnancy at the time of ART initiation impacted ART adherence or virologic suppression during the first year of therapy [15]. ART-naive individuals over 18 years old were enrolled from publicly operated HIV clinics in Cape Town, South Africa and southwestern Uganda at the time of ART initiation, and observed at study visits again at 6 and 12 months. To investigate causes of missed study visits, study staff performed phone calls and, if participants were not reachable, home visits. Use of trimethoprim-sulfamethoxazole (TMP-SMX), which is recommended for all individuals on ART in Uganda and for those with a CD4^+^ cell count less than 200 cells/μl in South Africa, was collected via self-report in Uganda and extracted from available pharmacy databases in two of three study clinics in South Africa. Deaths were confirmed by verbal autopsy from household members, or when available, from review of medical records. Those missing from follow-up without a confirmed death were defined as lost from observation.

#### Laboratory methods

At each study visit, blood was collected into EDTA tubes and tested for CD4^+^ cell count (Pima, Abbott Laboratories, Lake Bluff, Illinois, USA) and HIV-1 RNA viral load [Cobas Taqman platform in Uganda (Applied Biosciences, Beverley Hills, California, USA) and the CAP/CTM HIV-1 v2 assay (Roche Laboratories, Basel, Switzerland) in South Africa]. Additional blood was centrifuged for plasma separation, and frozen at -80^o^C. Due to resource constraints that prevented us from testing the entire cohort, only nonpregnant individuals underwent additional testing of cryopreserved plasma for interleukin (IL-6; MesoScale Discovery, Rockville, Maryland, USA); soluble CD14 (sCD14; R&D Systems, Minneapolis, Minnesota, USA); and D-dimer (Diagnostica Stago, Parsippany, New York, USA). Biomarker assays were performed at the Laboratory for Clinical Biochemistry Research at the University of Vermont.

#### Statistical methods

We used survival analysis methods with a primary outcome of all-cause mortality and a secondary outcome of a composite of mortality or loss from observation. Participants were censored on their 12-month visit, their date of death (for mortality) or on their last contact with study staff (for those lost from observation). We first used Kaplan–Meier methods to graphically depict mortality and mortality/loss over observation time by tertiles of each biomarker. We then estimated crude mortality per 100 person-years by tertile of each biomarker. After noting minimal differences in mortality rates between the lowest and middle tertiles of biomarkers, we combined these two groups in regression models. We fit Cox proportional hazards models for both outcomes, both with and without additional predictors of mortality in the model, including age, sex, active smoking, pretreatment CD4^+^ cell count and pretreatment log_10_ HIV-1 RNA viral load. We fit univariable models stratified by sex, CD4^+^ cell count (>350 versus <200 cells/μl), and recorded use (or not) of TMP-SMX for opportunistic infection prophylaxis among those who met criteria, and tested for an interaction term between these subgroups and biomarker category subgroups. We graphed log-log plots of survival versus observation time to assess for the proportional hazards assumption in both the mortality and the mortality/loss from observation models. Finally, we fit receiver operator curves for each biomarker and CD4^+^ cell count as predictors of mortality in the first 12 months of observation, and compared the area under the receiver operator curve (AUROC) of each of the biomarkers to CD4^+^ cell count using chi-squared testing.

#### Ethical considerations

Study procedures were approved by Partners Healthcare, the Mbarara University of Science and Technology, Uganda National Council for Science and Technology, University of Cape Town and Western Cape province in South Africa. All participants provided written informed consent.

## Results

Six hundred and sixty out of 699 (95%) individuals were enrolled in the META study, completed pretreatment biomarker testing and had a detectable viral load at baseline. Approximately 60% of participants were women, with a median age of 33 years, a median CD4^+^ cell count of 187 cells/μl, and the cohort was nearly evenly split between individuals from Uganda and South Africa (Supplemental Table 1). When we divided between individuals in the lower two tertiles and the highest tertile of pretreatment sCD14, those in the highest tertile had higher pretreatment HIV-1 RNA viral load and lower median CD4^+^ cell count.

**Fig. 1:**
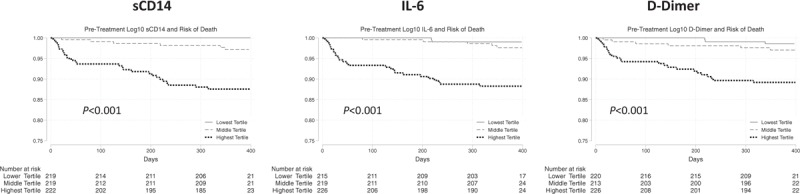
Pretreatment inflammatory markers and risk of mortality.

A total of 34 people (5.2%) died during observation for a crude mortality incidence of 5.3 per 100 person-years (py) [95% confidence interval (95% CI) 3.8–7.4]. An additional 12 participants (1.8%) were lost from observation. We found significant differences in mortality by tertile of pretreatment biomarkers (all *P* ≤ 0.001, Supplemental Table 2, Fig. 1). For example, the mortality rates were 0/100 py and 13.7/100py (9.4–20.0/100 py) comparing the low with the highest tertiles of sCD14 (Supplemental Table 2). The survival curves were similar when selecting a composite of death or loss from observation as the outcome of interest (Supplemental Figure 1).

In unadjusted Cox proportional hazards models, pretreatment HIV-1 RNA viral load (hazard ratio 2.05, 95% CI 1.35–3.12, *P* = 0.001) and CD4^+^ cell count (hazard ratio 0.57 for each 100 cells/μl, 95%CI 0.44–0.75, *P* < 0.001), and each of the three biomarkers were associated with hazard of mortality (Supplemental Table 3). These patterns were similar when selecting mortality or loss from observation as the outcome of interest (Supplemental Table 4). In adjusted models, those with the highest versus the two lowest tertiles of all three biomarkers prior to treatment remained significantly associated with hazard of mortality [sCD14: adjusted hazard ratio (AHR) 5.83, 95% CI 2.19–15.34, *P* < 0.001; IL6: AHR 4.65, 95% CI 1.90–11.36, *P* = 0.001; D-dimer: AHR 2.87, 95% CI 1.32–6.29, *P* = 0.01]. In contrast, the effect size of both pretreatment viral load and CD4^+^ cell count diminished after addition of biomarkers to multivariable models, and all lost significance, aside from CD4^+^ cell count in the D-dimer model. Moreover, the hazard of mortality for the lower two versus the highest tertile biomarkers was similar in those with low and high CD4^+^ cell counts (<200 versus >350 cells/μl) and in both men and women, with interaction terms for all subgroup effects (*P* > 0.20, Supplemental Figure 2). This finding was particularly notable for sCD14, in whom the hazard of mortality for those in the highest tertile was similar for those with a CD4^+^ cell count less than 200 and more than 350 cells/μl (6.28, 95% CI 2.19–18.01 versus 7.06, 95% CI 1.00–50.2, *P* value for interaction term = 0.95). Seventy-eight percent (354/453) of those meeting criteria had recorded use of TMP-SMX at enrolment. The crude mortality rate was significantly higher in those who did not receive TMP-SMX (15.8 deaths/100 py versus 3.2 deaths/100 py, *P* < 0.001). Although the predictive nature of the biomarkers was somewhat diminished, we found no significant differences in the prognostic value of the biomarkers in those who did versus did not have TMP-SMX use recorded (Supplemental Table 5, Supplemental Figure 2).

**Fig. 2:**
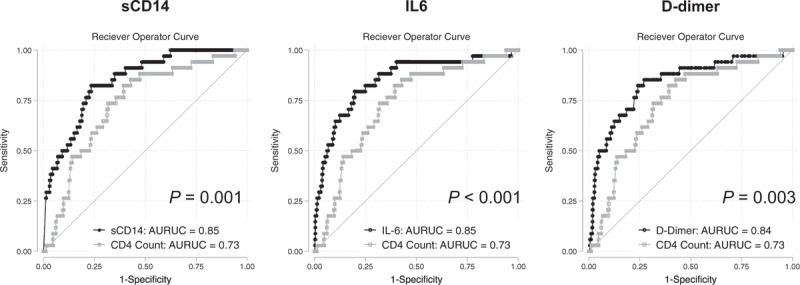
Receiver operator curves for prediction of all-cause mortality in first 12 months of therapy for pretreatment soluble biomarkers of inflammation, immune activation and coagulation versus pretreatment CD4^+^ cell count.

Log-log plots of the inflammatory markers demonstrated largely parallel plots consistent with the proportional hazard assumption (Supplemental Figure 3). Finally, we found that the AUROC for prediction of 12-month mortality was higher for each of the three biomarkers than for CD4^+^ cell count [sCD14: 0.85 (0.7–0.91); IL6: 0.85 (0.78–0.92); D-dimer: 0.84 (0.76–0.91); CD4^+^ cell count: 0.74 (0.66–0.81); *P* value for all comparisons versus CD4^+^ cell count <0.01, Fig. 2].

## Discussion

Herein, we demonstrate that biomarkers of immune activation, systemic inflammation and coagulopathy are highly predictive of mortality in the first 12 months of HIV therapy in South Africa and Uganda, and might serve as efficient prognostic indicators for patients initiating therapy. All three biomarkers evaluated had higher discriminatory prediction of 12-month mortality than CD4^+^ cell count based on AUROC and each remained significantly predictive of mortality in people with low CD4^+^ cell counts. For example, those in the highest tertile of sCD14 prior to ART had approximately a seven-fold increase in mortality than those in the two lowest tertiles, irrespective of CD4^+^ cell count, and the mortality rate in the lowest versus high tertile was 0/100py versus 13.7/100py. Moreover, in adjusted models, the biomarkers remained predictive of mortality, whereas both pretreatment CD4^+^ cell count and pretreatment viral load had much lower and largely nonsignificant effect sizes.

There has been strong interest in scalable strategies to differentiate HIV care in SSA, where there are goals to retain over 25 million people in chronic care [9]. Because such programmes focus on ‘stepped-down care’ for stable and low-risk care, a critical element of their success is an ability to accurately identify patients at greatest risk for opportunistic infections and early mortality. In practice, most programmes rely upon CD4^+^ cell count to make determinations about risk of opportunistic infections. Yet, our data suggest that certain biomarkers, such as D-dimer and sCD14, might better discriminate between those with the highest risk of mortality. A critical next step will be to determine whether such biomarkers can also triage high-risk patients for additional testing and/or closer observation to ultimately improve outcomes.

Multiple prior studies have investigated the use of a variety of other prognostic indicators, such as albumin and blood indices, for identifying those at risk of early mortality [16,17]. Although many of these studies have found markers associated with death, they largely demonstrate adjusted effect sizes for increased odds or hazard of mortality in the range of 10–30%. In contrast, both in this analysis and in prior studies in the region [11], pretreatment biomarkers of inflammation and systemic inflammation appear to predict a 300–500% increased risk of early mortality. These effect sizes are similar or larger than other frequently used biomarkers for disease prognostication. For example, similar differences in mortality of two-fold to four-fold and AUROC in the range of 0.6–0.8 are characteristic of scores to predict in-hospital mortality with sepsis [18]. Importantly, point-of-care assays for D-dimer and sCD14 have been developed and validated, and thus could be potentially adopted as scalable and clinically implementable assays if their prognostic value can be corroborated [19,20].

Our study was limited by the use of retrospective testing of cryopreserved plasma for biomarkers in a central laboratory, which prevents us from extrapolating our results into clinical practice. We also lacked data on incident opportunistic infections or adjudicated causes of death, which were not within the scope of the parent study, so we could not directly determine whether the biomarkers examined were efficient surrogates of preventable causes of death. A strength of our study was near complete assessment of survival, with only 12 (2%) of participants lost from observation, and models that allocated these individuals as failures did not substantially affect our results. Future work should evaluate the use of biomarkers to triage patients at high-risk for adverse clinical outcomes in early treatment. A particularly important question is whether identification of such patients, and providing them with enhanced clinical oversight, can result in lower mortality rates. If so, and if such biomarkers can be made available at cost and scale, they could serve a key role in promotion of differentiated models of care to optimize use of scarce resources as HIV treatment programmes scale in SSA.

## Acknowledgements

We thank all study participants for the involvement in the study, and the study staff, including *Research Assistants*: Nomakhaya April (RN), Alienah Mpahleni, Vivie Situlo, Speech Mzamo, Nomsa Ngwenya, Khosi Tshangela Regina Panda, Teboho Linda, Christine Atwiine, Sheila Moonight, Edna Tindimwebwa, Nicholas Mugisha, Peace Atwogeire, Dr Vian Namana, Catherine Kyampaire, Gabriel Nuwagaba; *Program Managers*: Annet Kembabazi, Stephen Mugisha, Victoria Nanfuka, Anna Cross, Nicky Kelly, Daphne Moralie, Kate Bell; *Statistician*: Nicholas Musinguzi; *Data Managers*: Dolphina Cogill, Justus Ashaba, Zoleka Xapa, Mathias Orimwesiga, Elly Tuhanamagyezi, Catherine Kyampaire; *Lab Managers*: Don Bosco Mpanga, Leonia Kyarisima, Simone Kigozi.

The META Study Team includes the following scientific investigators: Principal Investigators: Dr Jessica Haberer, Dr Catherine Orrell, Dr Norma Ware, Dr Mwebesa Bosco Bwana, Dr Asiimwe Stephen, Dr Amanyire Gideon, Hon. Dr Tumwesigye Elioda, Dr David Bangsberg; Co-Investigators: Dr Alexander C. Tsai, Dr Mark Siedner, Dr Lynn Matthews, Dr Ingrid Katz, Monique Wyatt.

This work was supported by the Bill and Melinda Gates Foundation (OPP113634). The authors acknowledge the following additional sources of support from the National Institutes of Health including K23 MH099916, P30 AI060354 and R01 HL141053.

### Conflicts of interest

All authors report no conflicts of interest

## Supplementary Material

Supplemental Digital Content
