# Supplementary material for: Inflammatory biomarkers prior to antiretroviral therapy as prognostic markers of 12-month mortality in South Africa and Uganda
Source: AIDS. 2019 Jul 2;33(13):2043–8. doi: 10.1097/QAD.0000000000002305 (PMC6774817; doi:10.1097/QAD.0000000000002305)
Supplement: Supplemental Digital Content [file aids-33-2043-s001.doc]

**Supplemental Table 1**. Cohort Characteristics

| **Characteristic** | **Total Cohort (n=660)** | **Lower Two Quartiles of sCD14 (n=438)** | **Highest Tertile of Pre-Treatment sCD14 (n=22)** | ***P*-value** |
| --- | --- | --- | --- | --- |
| Median Age (IQR) | 33 (27 – 41) | 33 (27 – 40) | 33 (28 – 41) | 0.25 |
| Female (n, %) | 396 (60) | 274 (63) | 122 (55) | 0.06 |
| Country |  |  |  |  |
| Uganda vs South Africa (n, %) | 321 (49) | 202 (46) | 119 (54) | 0.07 |
| Current Smoker (n, %) | 63 (10) | 43 (10) | 20 (9) | 0.26 |
| Log 10 Viral Load in copies/mL  (median, IQR) | 4.7 (4.1 – 5.2) | 4.4 (3.6 – 4.9) | 5.2 (4.7 – 5.6) | <0.001 |
| CD4 count in cells/uL  (median, IQR) | 187 (111 – 425) | 380 (157 – 444) | 120 (55 – 177) | <0.001 |

*P*-value results represent statistical tests of comparison between those with lower and highest quartile of soluble CD14 at the pre-treatment visit, performed with rank-sum testing for continuous variables and chi-squared testing for categorical or dichotomous variables.

sCD14: Soluble CD14; ART: antiretroviral therapy; IQR: interquartile range

**Supplemental Table 2**. Incidence of mortality by pre-treatment tertile of inflammatory markers

|  | Median Value (IQR) | Person-Years of Observation | Deaths | Mortality Rate  (per 100 person-years) |
| --- | --- | --- | --- | --- |
| Pre-Treatment Soluble CD14 |  |  |  |  |
| Lowest Tertile | 1040 (920, 1141) | 218.2 | 0 | -- |
| Middle Tertile | 1408 (1307, 1517) | 217.0 | 6 | 2.7 (1.2, 6.2) |
| Highest Tertile | 2120 (1853, 2479) | 204.8 | 28 | 13.7 (9.4, 20.0) |
| Pre-Treatment Interleukin 6 |  |  |  |  |
| Lowest Tertile | 0.44 (0.34, 0.54) | 214.4 | 2 | 0.9 (0.0, 4.0) |
| Middle Tertile | 0.95 (0.79, 1.18) | 218.3 | 5 | 2.2 (1.0, 5.5) |
| Highest Tertile | 3.54 (2.17, 6.30) | 207.4 | 27 | 13.0 (8.9, 19.0) |
| Pre-Treatment D-Dimer |  |  |  |  |
| Lowest Tertile | 0.23 (0.15, 0.29) | 221.6 | 3 | 1.4 (0.4, 4.2) |
| Middle Tertile | 0.58 (0.48, 0.71) | 207.7 | 7 | 3.4 (1.6, 7.1) |
| Highest Tertile | 1.70 (1.15, 2.78) | 209.8 | 24 | 11.4 (7.7, 17.1) |

**Supplemental Table 3**. Cox proportional hazards model of hazard of mortality after antiretroviral therapy initiation

|  |  | | **Soluble CD14** | | **IL-6** | | **D-Dimer** | |
| --- | --- | --- | --- | --- | --- | --- | --- | --- |
|  | Univariable Models | | Multivariable Model | | Multivariable Model | | Multivariable Model | |
|  | HR (95%CI) | *P*-value | AHR (95%CI) | *P*-value | AHR (95%CI) | *P*-value | AHR (95%CI) | *P*-value |
| Age (each 10 years) | 1.20  (0.86, 1.67) | 0.27 | 1.16  (0.83, 1.62) | 0.37 | 1.19  (0.85, 1.67) | 0.30 | 1.20  (0.84, 1.67) | 0.29 |
| Female | 0.66  (0.34, 1.29) | 0.22 | 0.83  (0.41, 1.67) | 0.59 | 0.86  (0.42, 1.72) | 0.65 | 0.77  (0.38, 1.55) | 0.46 |
| South Africa  (vs Uganda) | 0.93  (0.47, 1.82) | 0.83 | 1.00  (0.49, 2.02) | 0.99 | 0.88  (0.44, 1.76) | 0.71 | 0.88  (0.44, 1.76) | 0.72 |
| Current Smoking | 0.58  (0.14, 2.42) | 0.45 | 0.50  (0.11, 2.22) | 0.36 | 0.47  (0.11, 2.07) | 0.32 | 0.43  (0.10, 1.91) | 0.27 |
| Pretreatment VL  (Log10 copies/mL) | **2.05**  **(1.35, 3.12)** | **0.001** | 1.11  (0.69, 1.78) | 0.68 | 1.27  (0.80, 2.00) | 0.31 | 1.28  (0.81, 2.04) | 0.30 |
| CD4 count  (each 100 cells/uL) | **0.57**  **(0.44, 0.75)** | **<0.001** | 0.75  (0.56, 1.02) | 0.06 | 0.75  (0.55, 1.01) | 0.06 | **0.69**  **(0.51, 0.93)** | **0.01** |
| **Log10 sCD14** |  |  |  |  |  |  |  |  |
| 1^st^ and 2^nd^ Tertiles | REF | -- | REF | -- |  |  |  |  |
| 3^rd^ Tertile | **9.64**  **(3.99, 23.30)** | **<0.001** | **5.83**  **(2.19, 15.54)** | **<0.001** |  |  |  |  |
| **Log10 IL6** |  |  |  |  |  |  |  |  |
| 1^st^ and 2^nd^ Tertiles | REF | -- |  |  | REF | -- |  |  |
| 3^rd^ Tertile | **7.86**  **(3.42, 18.05)** | **<0.001** |  |  | **4.65**  **(1.90, 11.37)** | **0.001** |  |  |
| **Log10 D-Dimer** |  |  |  |  |  |  |  |  |
| 1^st^ and 2^nd^ Tertiles | REF | -- |  |  |  |  | REF | -- |
| 3^rd^ Tertile | **4.80**  **(2.20, 10.04)** | **<0.001** |  |  |  |  | **2.87**  **(1.32, 6.29)** | **0.01** |

AHR: adjusted hazard ratio; HR: hazard ratio; REF: reference category; VL: viral load

**Supplementary Table 4**. Cox proportional hazards model of hazard of mortality or loss from observation after antiretroviral therapy initiation

AHR: adjusted hazard ratio; HR: hazard ratio; REF: reference category; VL: viral load

|  |  | | **Soluble CD14** | | **IL-6** | | **D-Dimer** | |
| --- | --- | --- | --- | --- | --- | --- | --- | --- |
|  | Univariable Models | | Multivariable Model | | Multivariable Model | | Multivariable Model | |
|  | HR (95%CI) | *P*-value | AHR (95%CI) | *P*-value | AHR (95%CI) | *P*-value | AHR (95%CI) | *P*-value |
| Age (each 10 years) | 1.13  (0.84, 1.50) | 0.42 | 1.08  (0.81, 1.45) | 0.60 | 1.11  (0.83, 1.49) | 0.47 | 1.11  (0.83, 1.50) | 0.46 |
| Female | 0.85  (0.48, 1.53) | 0.59 | 0.96  (0.52, 1.78) | 0.91 | 0.99  (0.54, 1.83) | 0.98 | 0.94  (0.51, 1.73) | 0.83 |
| South Africa  (vs Uganda) | 1.21  (0.68, 2.18) | 0.52 | 1.29  (0.70, 2.38) | 0.42 | 1.12  (0.61, 2.05) | 0.72 | 1.14  (0.63, 2.08) | 0.67 |
| Current Smoking | 0.65  (0.20, 2.09) | 0.47 | 0.55  (0.16, 1.89) | 0.34 | 0.54  (0.16, 1.84) | 0.34 | 0.52  (0.15, 1.77) | 0.30 |
| Pretreatment VL  (Log10 copies/mL) | **1.78**  **(1.26, 2.53)** | **0.001** | 1.16  (0.78, 1.75) | 0.45 | 1.33  (0.90, 1.96) | 0.16 | 1.33  (0.90, 1.98) | 0.15 |
| CD4 count  (each 100 cells/uL) | **0.72**  **(0.59, 0.87)** | **0.001** | 0.90  (0.73, 1.10) | 0.27 | 0.86  (0.69, 1.07) | 0.19 | 0.83  (0.67, 1.03) | 0.09 |
| **Log10 sCD14** |  |  |  |  |  |  |  |  |
| 1^st^ and 2^nd^ Tertiles | REF | -- | REF | -- |  |  |  |  |
| 3^rd^ Tertile | **4.76**  **(2.53, 8.92)** | **<0.001** | **3.57**  **(1.34, 8.93)** | **0.001** |  |  |  |  |
| **Log10 IL6** |  |  |  |  |  |  |  |  |
| 1^st^ and 2^nd^ Tertiles | REF | -- |  |  | REF | -- |  |  |
| 3^rd^ Tertile | **3.50**  **(1.90, 6.36)** | **<0.001** |  |  | **2.42**  **(1.26, 4.67)** | **0.008** |  |  |
| **Log10 D-Dimer** |  |  |  |  |  |  |  |  |
| 1^st^ and 2^nd^ Tertiles | REF | -- |  |  |  |  | REF | -- |
| 3^rd^ Tertile | **2.85**  **(1.59, 5.13)** | **<0.001** |  |  |  |  | **1.97**  **(1.05, 3.72)** | **0.04** |

**Supplemental Table 5**. Hazard Ratio of Mortality for highest (versus two lowest) tertiles of biomarkers in those with and without recorded use of trimethoprim-sulfamethoxazole prophylaxis

|  | Recorded Use of TMP-SMX | | No Recorded Use of TMP-SMX | |  |
| --- | --- | --- | --- | --- | --- |
|  | HR (95%CI) | P-value | HR (95%CI) | P-value | Interaction Term P-Value |
| sCD14 | 3.87  (1.07 – 13.92) | 0.04 | 7.48  (1.60 – 34.91) | 0.01 | 0.49 |
| IL6 | 5.06  (1.33 – 19.21) | 0.02 | 7.40  (1.65 – 33.06) | 0.01 | 0.74 |
| D-Dimer | 3.19  (0.93 – 11.00) | 0.07 | 2.86  (1.17 – 7.03) | 0.02 | 0.95 |
